# Amiodarone-Induced Pulmonary Toxicity Presenting as Hypoxemic Respiratory Failure in a Patient With Ventricular Tachycardia: A Case Report

**DOI:** 10.7759/cureus.83564

**Published:** 2025-05-06

**Authors:** Ammar Al Heyasat, Maida S Chaudhry, Ajwah Qasim, Zamir Shaikh

**Affiliations:** 1 Internal Medicine, Crestwood Medical Center, Huntsville, USA; 2 Internal Medicine, DHR Health Institute For Research And Development, Edinburg, USA

**Keywords:** amiodarone-induced pulmonary toxicity, antiarrhythmic therapy, bronchoalveolar lavage (bal), corticosteroids, drug-induced lung injury, high-resolution computed tomography (hrct), hypoxemic respiratory failure, implantable cardioverter-defibrillator (icd), organizing pneumonia, ventricular tachycardia

## Abstract

Amiodarone-induced pulmonary toxicity (APT) is a potentially life-threatening adverse effect of a commonly prescribed antiarrhythmic agent. APT can present with a wide spectrum of clinical and radiologic manifestations, often mimicking infectious or interstitial lung diseases, making timely diagnosis a clinical challenge. We report the case of a 54-year-old male with a history of ventricular tachycardia (VT) and an implantable cardioverter-defibrillator (ICD), who presented with progressive dyspnea and hypoxemic respiratory failure three months after the initiation of oral amiodarone therapy. Imaging revealed diffuse bilateral pulmonary infiltrates, and infectious and cardiac etiologies were excluded through bronchoscopy, bronchoalveolar lavage, and cardiac evaluation. High-resolution computed tomography (HRCT) demonstrated features consistent with organizing pneumonia. Following the prompt discontinuation of amiodarone and the initiation of systemic corticosteroids, the patient exhibited marked clinical and radiological improvement. This case underscores the importance of maintaining a high index of suspicion for APT in patients on long-term amiodarone therapy presenting with new respiratory symptoms.

## Introduction

Amiodarone is a highly effective class III antiarrhythmic agent used for ventricular and supraventricular arrhythmias [1]. However, its use is limited by serious adverse effects, including pulmonary toxicity (APT), which occurs in 1-5% of patients and can be life-threatening [2]. APT presents with nonspecific respiratory symptoms, often mimicking infections, making early diagnosis challenging [2]. We present a case of amiodarone-induced pulmonary toxicity to highlight the importance of prompt recognition and management in patients developing unexplained dyspnea or radiographic lung abnormalities during therapy.

## Case presentation

**Figure 1:**
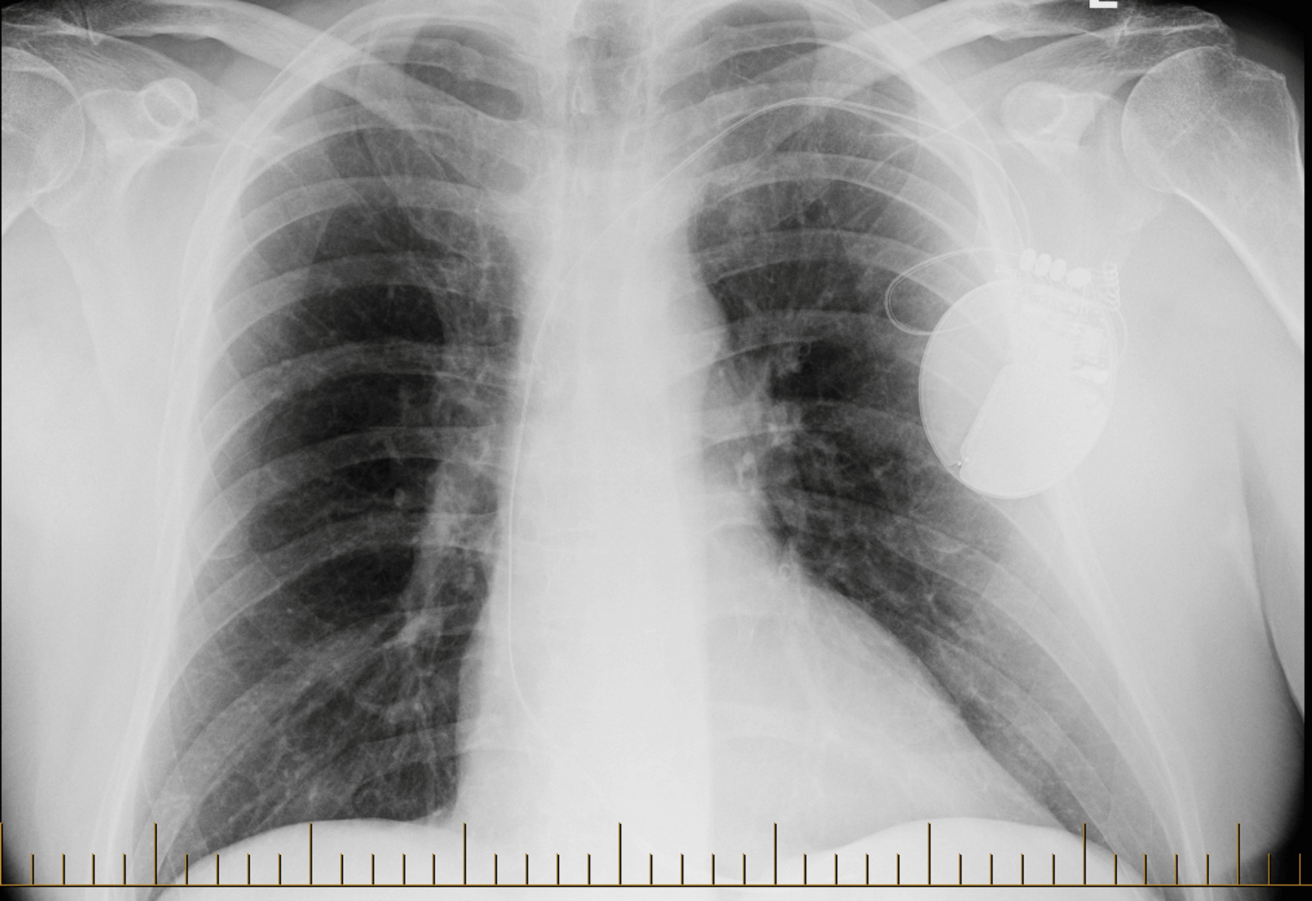
Posteroanterior (PA) chest X-ray showing no acute cardiopulmonary findings The imaging demonstrates a single-lead left subclavian implantable cardioverter-defibrillator (ICD) in place, with stable positioning and no signs of lead displacement or complications.

A 54-year-old male with a history of type 2 diabetes mellitus and anxiety disorder presented to the emergency department with progressive shortness of breath and a nonproductive cough of three weeks' duration. His past cardiac history included myocardial infarction in 2006, recurrent episodes of ventricular tachycardia requiring three ablations (most recent in 2021), and implantable cardioverter-defibrillator (ICD) placement in 2019. Due to frequent episodes of ventricular tachycardia (VT), the patient had been initiated on oral amiodarone three months prior to admission, starting with a loading dose of 400 mg twice daily for two weeks, followed by a maintenance dose of 200 mg twice daily. He was also taking sotalol 160 mg twice daily and metoprolol succinate 50 mg twice daily. Six months before admission, the patient presented with flu-like symptoms and underwent a chest X-ray, which showed no acute cardiopulmonary pathology (Figure 1).

He recovered without complications. However, in the weeks leading to his current admission, he developed progressive exertional dyspnea. On arrival at the emergency department, his oxygen saturation was found to be 76% on room air, prompting immediate placement on bilevel positive airway pressure (BIPAP). He was admitted to the intensive care unit (ICU) for further management.

**Figure 2:**
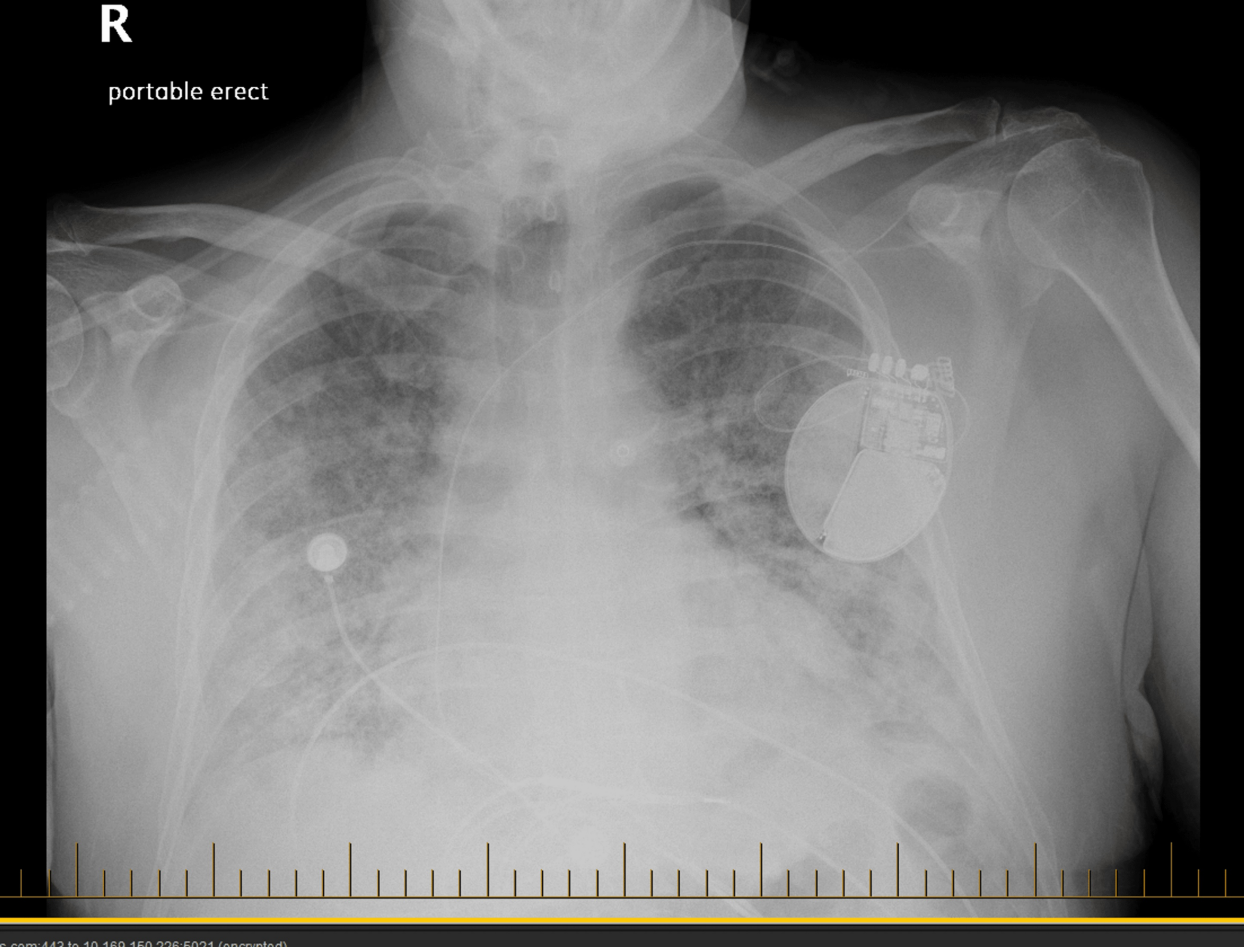
Portable erect chest X-ray demonstrating diffuse patchy opacities bilaterally, which appear unchanged from prior imaging No evidence of pleural effusion or pneumothorax is noted. The left chest wall pacemaker lead tips remain in a stable position.

Upon admission, the patient was hemodynamically stable. Vital signs were as follows: temperature 37.1 °C, heart rate 92 beats per minute, respiratory rate 28 breaths per minute, and blood pressure 138/82 mmHg. Oxygen saturation was 76% on room air. Arterial blood gas (ABG) analysis on room air revealed a pH of 7.49, partial pressure of carbon dioxide (PaCO₂) of 29 mmHg, partial pressure of oxygen (PaO₂) of 58 mmHg, bicarbonate (HCO₃⁻) of 26 mEq/L, and an oxygen saturation of 72%. A 12-lead ECG demonstrated normal sinus rhythm (heart rate 92 bpm), PR interval 160 ms, QRS duration 88 ms, QT interval 420 ms, and no ST-segment deviations or T-wave inversions. A chest X-ray showed diffuse patchy bilateral opacities (Figure 2).

Laboratory evaluation showed a mild leukocytosis with a white blood cell count of 11.2 ×10³/µL. Hemoglobin was 13.4 g/dL, and hematocrit was slightly below normal at 40.2%. Platelet count was within normal limits at 210 ×10³/µL. Electrolytes, renal function, and liver enzymes were within normal limits. Notably, lactic acid was 1.4 mmol/L, indicating no significant lactic acidosis. A viral polymerase chain reaction (PCR) panel was also performed on initial presentation, which was negative for common respiratory viruses, including COVID-19, influenza A, influenza B, influenza C, and respiratory syncytial virus (RSV).

**Figure 3:**
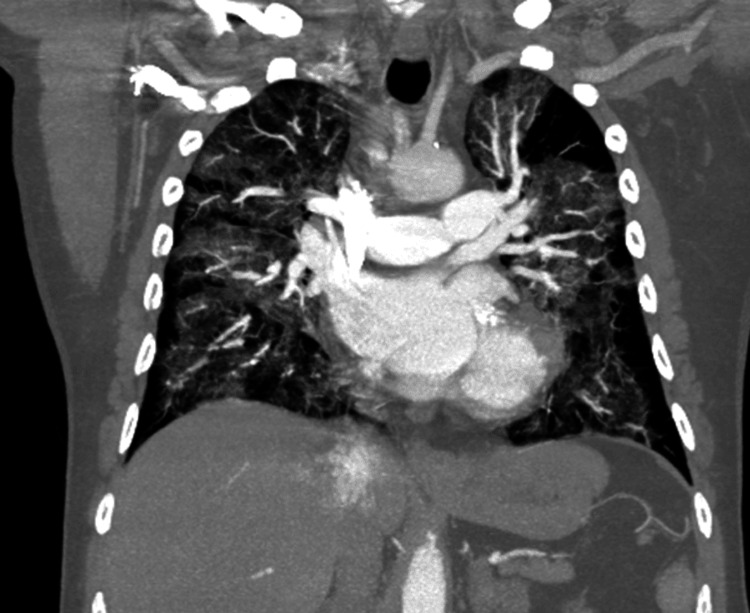
A coronal computed tomography (CT) angiogram of the chest was negative for pulmonary embolism; however, it revealed diffuse bilateral interstitial and airspace opacities

**Figure 4:**
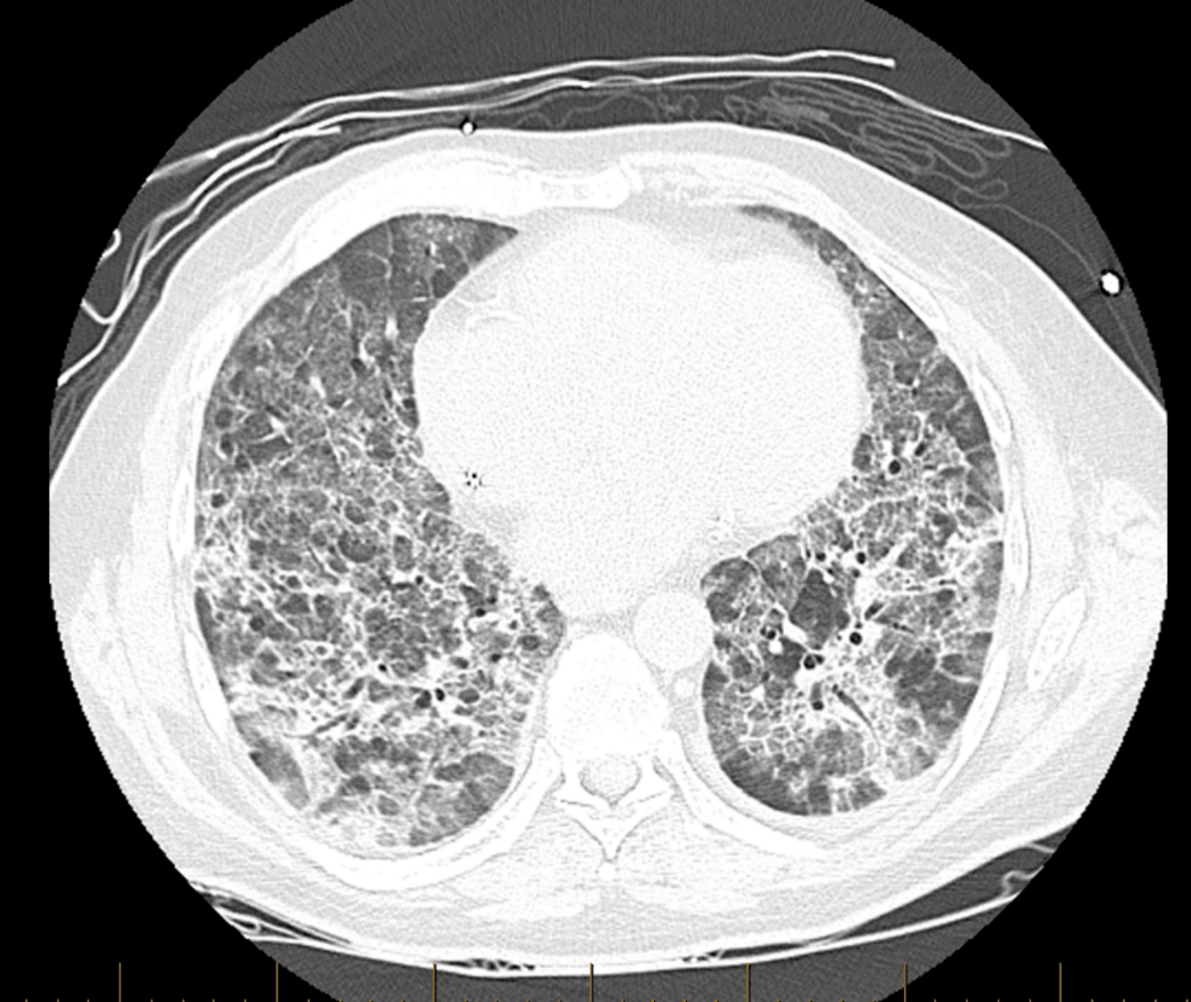
Axial non-contrast high-resolution computed tomography (HRCT) of the chest performed one week into hospitalization, demonstrating worsening lower lobe opacities, interlobular septal thickening, and new mild cylindrical bronchiectasis, with findings suggestive of possible early fibrosis

On admission, a contrast-enhanced computed tomography (CTA) scan of the chest ruled out pulmonary embolism but demonstrated bilateral diffuse interstitial and airspace opacities (Figure 3). A non-contrast high-resolution computed tomography (HRCT) chest, performed one week into hospitalization, revealed worsening lower lobe opacities, interlobular septal thickening, and new mild cylindrical bronchiectasis, with findings suggestive of possible early fibrosis (Figure 4).

**Figure 5:**
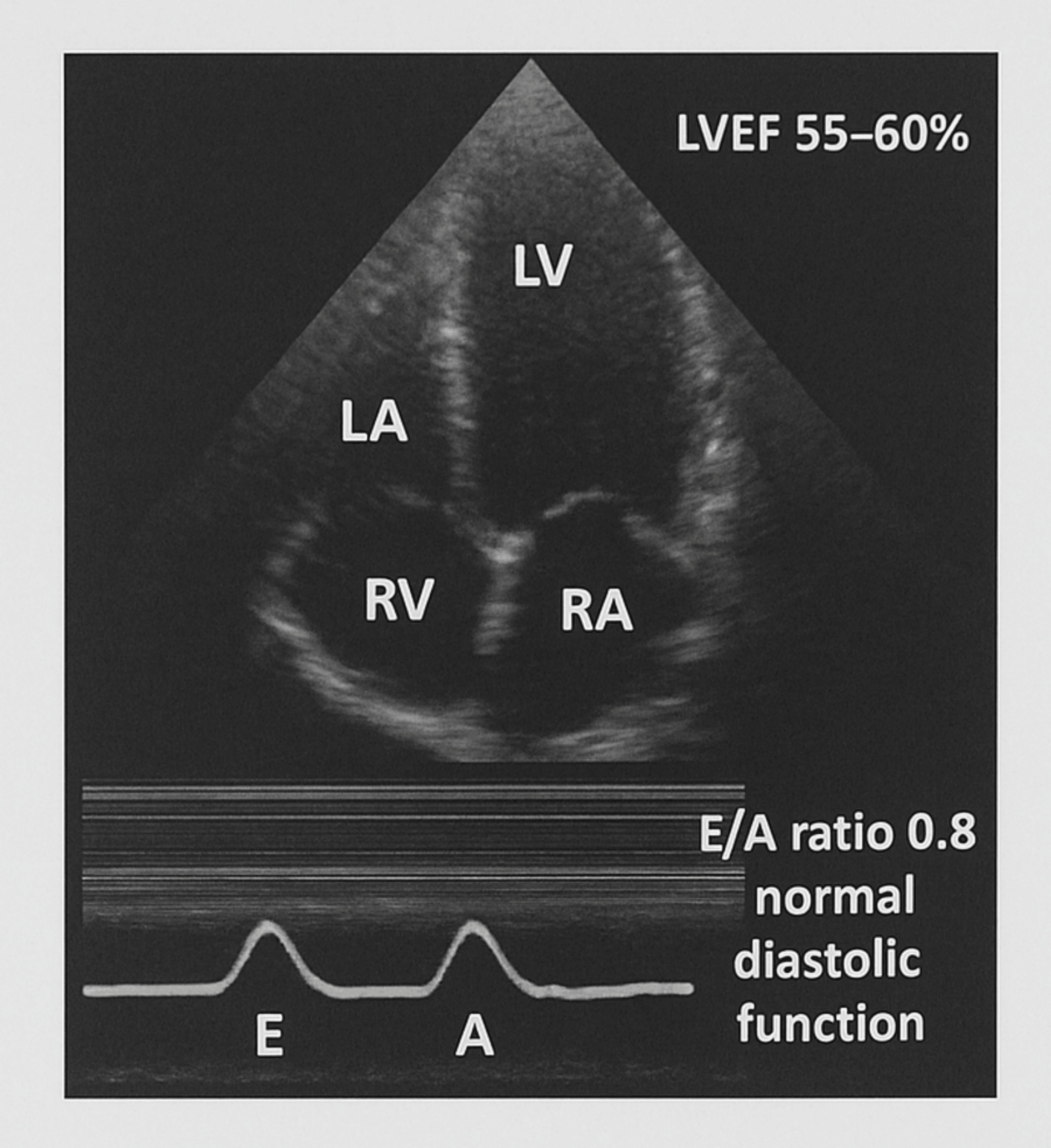
Apical four-chamber transthoracic echocardiogram The left ventricle (LV), right ventricle (RV), left atrium (LA), and right atrium (RA) are labeled. An inset M-mode tracing of mitral valve inflow demonstrates normal diastolic function (E/A ratio 0.8). The estimated left ventricular ejection fraction (LVEF) is 55–60%.

A transthoracic echocardiogram (Figure 5) demonstrated normal left ventricular dimensions and systolic function with an ejection fraction of 55-60%. Diastolic function was preserved with an E/A ratio of 0.8. All cardiac valves appeared structurally and functionally normal.

Bronchoalveolar lavage (BAL) analysis revealed no evidence of infection or malignancy. The Gram stain was negative for organisms, and bacterial cultures demonstrated no growth. Fungal cultures were also negative. Cytological examination showed no malignant cells, and testing for acid-fast bacilli was negative, suggesting no evidence of mycobacterial infection.

Thyroid function tests (thyroid-stimulating hormone (TSH) 1.8 µIU/mL, free T4 1.2 ng/dL), liver enzymes (aspartate aminotransferase (AST) 28 U/L, and alanine transaminase (ALT) 24 U/L), and ophthalmologic exam (no corneal deposits) showed no evidence of amiodarone toxicity. Dermatologic and neurologic exams were unremarkable. ICD interrogation was initially deferred due to respiratory compromise and was performed later, confirming no inappropriate shocks or arrhythmic burden. Upon suspicion of drug-induced lung injury, amiodarone was immediately discontinued. The patient was transitioned to sotalol 80 mg twice daily, with telemetry monitoring and serial ECGs to assess the QT interval. Broad-spectrum antibiotics, including cefepime and azithromycin, were initiated empirically, but these were discontinued after five days once all infectious workup, including BAL, returned negative.

**Figure 6:**
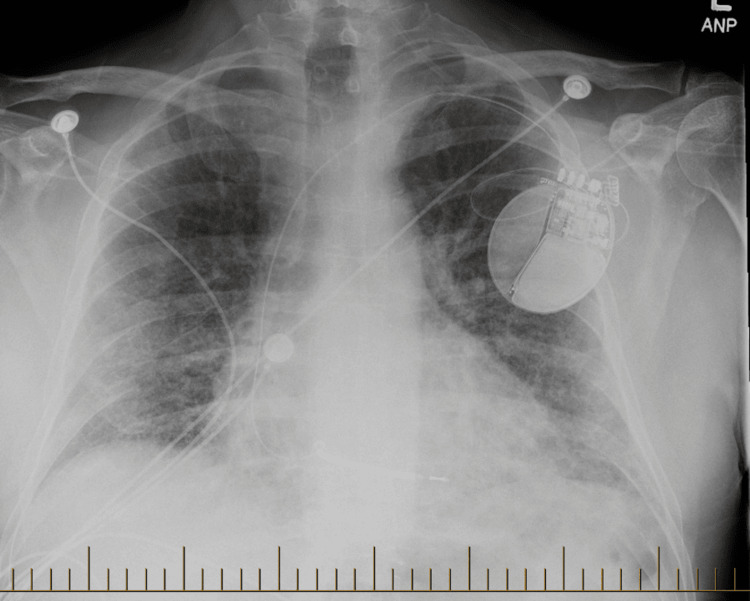
Follow-up chest X-ray obtained one month post-discharge, demonstrating bilateral improvement of pulmonary infiltrates

Given the severity of the patient’s hypoxemia and imaging findings, intravenous methylprednisolone 40 mg every 6 hours was initiated. The patient was initially downgraded from BiPAP to high-flow nasal cannula (HFNC), and over the following days, his oxygen requirements gradually decreased. When transitioned to a 3L nasal cannula, ABG analysis revealed a pH of 7.42, PaCO₂ of 38 mmHg, PaO₂ of 72 mmHg, HCO₃⁻ of 24 mEq/L, and oxygen saturation of 96%. The patient was discharged on a 3L nasal cannula and a tapering oral prednisone regimen. A follow-up chest X-ray one month post-discharge demonstrated significant bilateral improvement in pulmonary infiltrates (Figure 6). A detailed overview of the clinical course, including amiodarone dosing, imaging findings, and treatment interventions, is summarized in Table 1.

**Table 1:** Clinical timeline of amiodarone-induced pulmonary toxicity CXR: chest X-ray; VT: ventricular tachycardia; CTA: computed tomography angiography; BiPAP: bilevel positive airway pressure; ABG: arterial blood gas

Timepoint	Clinical Event	Amiodarone Dose	Imaging	Notes
-6 months	Baseline CXR (unrelated flu-like illness)	None	Normal CXR (Figure 1)	No pulmonary pathology
0 months	Amiodarone initiated	400 mg BID × 2 weeks → 200 mg BID maintenance	–	For recurrent VT
+2.5 months	Onset of symptoms (dyspnea, cough)	200 mg BID	–	Progressive worsening
+3 months	Hospital admission	200 mg BID	CXR: Bilateral opacities (Figure 2); CTA chest: Interstitial infiltrates (Figure 3); HRCT: Fibrotic changes (Figure 4)	Severe hypoxemia; BiPAP
Hospital Day 2	Amiodarone discontinued	Discontinued	–	Switched to sotalol
Hospital Day 3	IV steroids initiated	–	–	Methylprednisolone 40 mg Q6H
Hospital Day 7	HRCT repeated	–	Worsening opacities with bronchiectasis (Figure 4)	
Discharge	Tapered prednisone	–	ABG on 3L NC: pH 7.42, PaO₂ 72	Improved oxygenation
1-month follow-up	–	–	Chest X-ray: Resolution of infiltrates (Figure 6)	No recurrence of symptoms

## Discussion

Amiodarone-induced pulmonary toxicity (APT) is a serious adverse effect associated with amiodarone therapy, with an incidence ranging from 1% to 5% and a fatality rate of approximately 10% [1,2]. APT remains a diagnostic challenge due to its variable clinical and radiographic manifestations, which can range from mild interstitial pneumonitis to life-threatening acute respiratory distress syndrome (ARDS) [2,3]. Early identification is crucial, as delayed diagnosis and management can lead to irreversible pulmonary fibrosis or respiratory failure.

Clinical suspicion for APT should be high in patients on amiodarone who present with respiratory symptoms, such as cough, dyspnea, and hypoxemia, especially when imaging reveals bilateral pulmonary infiltrates and infectious etiologies have been excluded [2-5]. High-resolution CT findings may include ground-glass opacities, interstitial thickening, and organizing pneumonia patterns [4,5]. In our case, chest imaging demonstrated diffuse bilateral infiltrates, raising concern for APT.

Bronchoalveolar lavage (BAL) is a valuable tool in the diagnostic workup of APT. While BAL cytology findings are non-specific, they are crucial to exclude infectious causes and malignancy [2,3,6]. Cellular profiles can vary but often show a mixed inflammatory pattern. In our patient, infectious workup, including BAL analysis, was negative, supporting the diagnosis of APT.

The pathophysiology of APT involves both direct cytotoxic effects and immune-mediated mechanisms, leading to alveolar and interstitial inflammation [2,7]. Several risk factors, including cumulative amiodarone dose, duration of therapy, and pre-existing lung disease, have been identified [4,8]. However, APT can occur even at low doses and within a few months of therapy initiation, as illustrated in our case.

The cornerstone of APT management is prompt discontinuation of amiodarone and initiation of corticosteroid therapy. In our case, moderate-dose intravenous methylprednisolone was administered at 40 mg every six hours (totaling 160 mg/day), a regimen consistent with established approaches to drug-induced lung injury [2,3,5]. This treatment resulted in progressive clinical and radiographic improvement. Corticosteroid dosing in APT is generally guided by clinical severity, and typical regimens range from 0.5 to 1 mg/kg/day of prednisone or equivalent [2,5]. A gradual tapering schedule is recommended based on the patient’s response. Although no universally standardized duration exists, early intervention with corticosteroids has been associated with improved outcomes and reduced risk of irreversible pulmonary fibrosis [2,3].

Given the widespread use of amiodarone, heightened awareness and vigilance for APT are essential. Regular monitoring with chest imaging and pulmonary function tests, as well as early recognition of symptoms, can improve prognosis and reduce the risk of fatal complications [1,5,9,10].

This case emphasizes the need for high clinical suspicion for APT in patients presenting with respiratory symptoms while on amiodarone, particularly in the setting of bilateral infiltrates and a negative infectious workup. Timely recognition, drug cessation, and corticosteroid therapy can prevent progression to irreversible pulmonary fibrosis or respiratory failure. Given the potential for severe outcomes, including respiratory failure, early detection and intervention are critical.

## Conclusions

This case highlights the importance of maintaining a high index of suspicion for APT in patients receiving amiodarone who present with new respiratory symptoms. Early diagnosis, immediate cessation of amiodarone, and timely corticosteroid therapy are essential to halt disease progression and reduce the risk of permanent respiratory disability or death.
